# A new method for objective myocardial scar characterization using MR late gadolinium enhancement and post-contrast look-locker sequences

**DOI:** 10.1186/1532-429X-15-S1-P182

**Published:** 2013-01-30

**Authors:** Qian Tao, Hildo J Lamb, Katja Zeppenfeld, Rob J van der Geest

**Affiliations:** 1 Department of Radiology, Leiden University Medical Center, Leiden, Netherlands; 2 Department of Cardiology, Leiden University Medical Center, Leiden, Netherlands

## Background

Accurate characterization of myocardial scar has important diagnostic and prognostic implications for post-infarct patients. Previous studies have used late gadolinium enhanced (LGE) MR to characterize myocardial scar, by dividing the scar region into core and gray zones based on signal intensity in LGE. The characterization is however subjective to manual annotations, dependent on image acquisition parameters, contrast dose and timing, and has limited reproducibility. The purpose of this study is to objectively and reproducibly characterize infarcted myocardial tissue combining information from the LGE and post-contrast Look-Locker (LL) sequences.

## Methods

Eighty-four post-infarct patients underwent magnetic resonance imaging at 1.5 T. Prior to the LGE acquisition, a LL sequence was acquired at one short-axis level to determine the optimal inversion time for LGE, and to estimate the T1 values of blood and myocardium. In the training group of 52 patients, the blood pool, viable myocardium, and fibrotic tissue were manually annotated and their relaxation rates were derived from the T1 map. The relationship between the relaxation rates of the viable/fibrotic tissue and the blood was modeled by linear regression. In the testing group of 32 patients, the linear models were applied to estimate the relaxation rates of viable and fibrotic myocardial tissue. The T1-identified viable and fibrotic regions were projected from the T1 map to the corresponding LGE slice, and their signal intensity was used to estimate the percentage of infarction within the complete LV. The T1-based scar identification was compared to manual scar identification results from two independent observers. Reproducibility of our method was evaluated by comparing the T1-based scar identification results obtained by the two observers.

## Results

**Figure 1:**
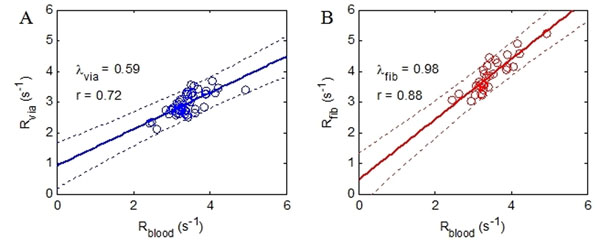
A. Linear regression between the post-contrast relaxation rate of the blood and viable tissue; B. Linear regression between the post-contrast relaxation rate of the blood and fibrotic tissue.

The relaxation rates of the viable/fibrotic tissue both exhibited a linear relationship (p<0.05, Figure 1) to that of blood. The slope of the linear fitting further indicated the blood-tissue partition coefficient in the viable/fibrotic tissue (λ=0.59 and 0.98, respectively). The T1-based scar identification showed good agreement with the manually identified scar regions, in terms of Dice index (0.90±0.06) and scar size relative to myocardium volume (32.6±11.2% vs. 32.7±12.0%, p=NS). Bland-Altman analysis showed that the inter-observer variability in scar identification was -2.8±3.6% for manual scar identification, and 0±1.3% for T1-based scar identification (p<0.05).

**Figure 2:**
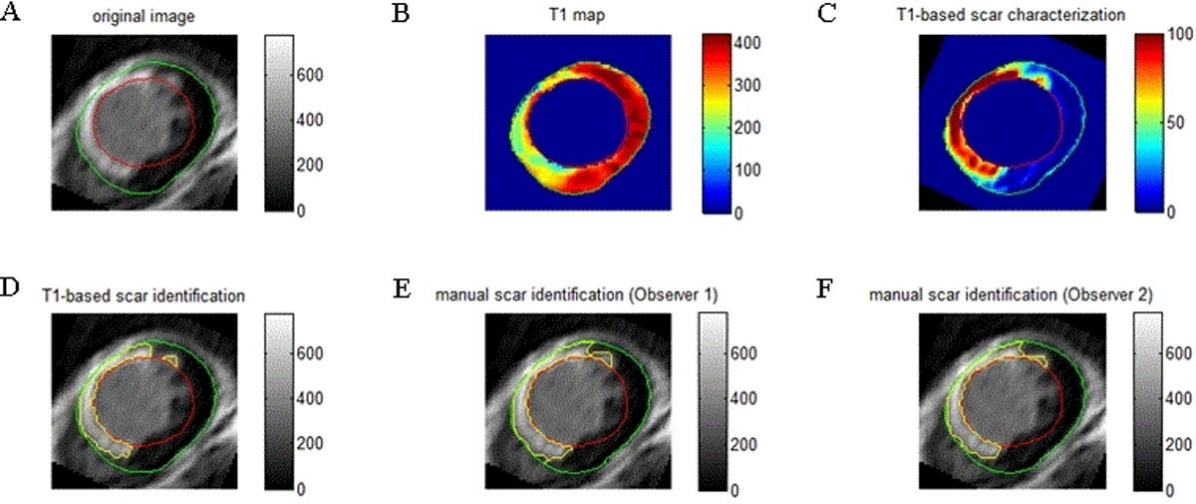
A. The original LGE image. B. The corresponding T1 map estimated from the post-contrast LL sequence. C. T1-based scar characterization in terms of percentage of infarction. D. T1-based scar region identification. E. Manual scar region identification from Observer 1. F. Manual scar region identification from Observer 2.

## Conclusions

By combining information from LL and LGE sequences, myocardial scar can be characterized in an objective and highly reproducible manner for post-infarct patients. Since the method does not require additional scanning it can be readily applied with standard cardiac CMR protocols.

## Funding

European MEDIATE project (ITEA 09039)

